# Improving the Acetic Acid Fermentation of *Acetobacter pasteurianus* by Enhancing the Energy Metabolism

**DOI:** 10.3389/fbioe.2022.815614

**Published:** 2022-03-08

**Authors:** Jia Song, Jun Wang, Xinyu Wang, Hang Zhao, Tao Hu, Zhiwei Feng, Zhi Lei, Weizhao Li, Yu Zheng, Min Wang

**Affiliations:** ^1^ State Key Laboratory of Food Nutrition and Safety, Key Laboratory of Industrial Fermentation Microbiology, Ministry of Education, College of Biotechnology, Tianjin University of Science and Technology, Tianjin, China; ^2^ Tian Di No. 1 Beverage Inc., Jiangmen, China

**Keywords:** *Acetobacter pasteurianus*, acetic acid fermentation, transcriptome, energy charge, energy metabolism

## Abstract

Energy metabolism is important for cell growth and tolerance against environment stress. In acetic acid fermentation by *Acetobacter pasteurianus*, the correlation coefficients of acid production rate with energy charge and ATP content were 0.9981 and 0.9826, respectively. The main energy metabolism pathway, including glycolysis pathway, TCA cycle, ethanol oxidation, pentose phosphate pathway, and ATP production, was constructed by transcriptome analysis. The effects of fermentation conditions, including dissolved oxygen, initial acetic acid concentration, and total concentration, on acetic acid fermentation and energy metabolism of *A. pasteurianus* were analyzed by using the RT-PCR method. The results showed the high energy charge inhibited glucose catabolism, and associated with the high ethanol oxidation rate. Consequently, a virtuous circle of increased ethanol oxidation, increased energy generation, and acetic acid tolerance was important for improving acetic acid fermentation.

## Introduction

Acetic acid is an important food material fermented from ethanol by using acetic acid bacteria (AAB) (Saichana et al., 2015). AAB can partially oxidize ethanol to acetic acid under the combined action of alcohol dehydrogenase (ADH) and acetaldehyde dehydrogenase (ALDH) with pyrroloquinoline quinone (PQQ) as coenzyme (Lynch et al., 2019). As the fermentation product, acetic acid shows strong toxicity to microorganism (Xia et al., 2016). It can pass through the cell membrane, increase the concentration of intracellular acetic acid, destroy some physiological functions of cells (Lasko et al., 2000; Peter et al., 2014; Trcek et al., 2015). Thus, acetic acid toxicity to AAB makes it difficult to achieve a highly efficient acetic acid fermentation (Yang et al., 2019). Several mechanisms reportedly help AAB resist acetic acid stress, relating to (I) PQQ-ADH (Trcek et al., 2006; Yakushi et al., 2018), (I) TCA cycle (Andrés-Barrao et al., 2012; Andrés-Barrao et al., 2016), (III) ATP binding cassette transporter, such as AatA (Nakano et al., 2006), (IV) amino acid metabolism (Yin et al., 2017), and (V) adaptive response of tolerant proteins (Ishikawa et al., 2010). Interestingly, all these mechanisms are related to energy metabolism or ATP. PQQ-ADH produces energy by oxidizing ethanol which is the main energy source for AAB in acetic acid fermentation. Combined with the ethanol respiration chain, intracellular H^+^ is pumped to the outside of the membrane, thus forming a proton gradient, which promotes adenosine triphosphatase (ATPase) to produce ATP for cell growth and metabolism (Qi et al., 2014a; Wang Z. et al., 2015). Besides ethanol, glucose is the other energy source in acetic acid fermentation, mainly through pentose phosphate (PPP) and glycolysis pathway and tricarboxylic acid (TCA) cycle (Azuma et al., 2009; Illeghems et al., 2013). ATP-dependent transporters AatA (Nakano et al., 2006)) and molecular chaperones DnaK and GroEL (Susin et al., 2006) protect cell against acetic acid often in an ATP-driven process. AAB can change energy metabolism by regulating related enzymes expression to adapt to acetic acid fermentation conditions, such as initial acetic acid (Zhang et al., 2017). The down-regulation of the TCA cycle blocks the oxidation of glucose to provide energy (Yang et al., 2019). Therefore, energy metabolism and energy status of AAB are important for acetic acid fermentation. Adenylate energy charge (EC) is an index used to measure the growth and energy status of microorganisms (Chapman et al., 1971; Guimaraes and Londesborough 2008).

Semi-continuous liquid submerged fermentation is widely used for industrial acetic acid production (De Ory et al., 2004; Krusong et al., 2015). Aeration, initial acetic acid concentration, and total concentration (the sum of ethanol and acetic acid concentrations) are the main factors affecting the efficiency of this process (De Ory et al., 2004; Zhou et al., 2009). Enhanced oxygen supply substantially increases enzyme activities during *Acetobacter pasteurianus* ethanol oxidation (Zheng et al., 2018a). When the dissolved oxygen (DO) concentration is maintained at 1–3 mg/L, the AAB have the largest specific growth rate (Romero et al., 1994). However, excessive aeration decreases the conversion ratio because of the volatilization of ethanol and acetic acid (Rubio-Fernández et al., 2004). A two-stage oxygen supply strategy was established to improve the acetic acid fermentation under the direction of energy metabolism framework (Zheng et al., 2018a). A certain amount of initial acetic acid can enhance ADH activity, and then promote acetic acid fermentation (De Ory et al., 2002; Xu et al., 2015). Ethanol is the carbon and energy source for AAB (Baena-Ruano et al., 2010). However, bacterial survival is affected when the ethanol concentration is above 48 g/L (Jiménez-Hornero et al., 2009). Therefore, the total concentration was considered to optimize the acetic acid fermentation (Krusong et al., 2015; Pothimon et al., 2020).

There are two energy sources, ethanol and glucose, in acetic acid fermentation. Oxidizing 1 mol of ethanol to acetic acid produces 493 kJ Gibbs free energy, whereas 1 mol glucose produces 2870 kJ Gibbs free energy (Adler et al., 2014). In the early stage of acetic acid fermentation, the glucose is oxidized to produce energy through the PPP pathway; in the middle stage the ethanol respiratory chain becomes the main pathway for energy supply; in the later stage the TCA cycle coupled with aerobic respiration pathway and the ethanol respiratory chain are responsible for the energy supply (Zheng et al., 2017; Zheng et al., 2018a). However, the effect of fermentation condition on the global energy metabolism and acetic acid formation is unclear. This work aimed to reveal the relationship between ethanol oxidation and energy generation of *A. pasteurianus*, and to improve the acetic acid fermentation according to the energy metabolism.

## Materials and Methods

### Strains and Media

Strain of *A. pasteurianus* CGMCC 3089 registered in the Chinese General Microbiological Culture Collection Center was used for the acetic acid fermentation. The strain was stored on solid medium containing 15 g/L of glucose, 10 g/L of yeast extract, 20 g/L of CaCO_3_, 28 g/L of ethanol, and 17 g/L of agar at 4°C. The seed medium contains 20 g/L of glucose, 15 g/L of yeast extract, and 28 of g/L ethanol. The fermentation medium contains 20 of g/L glucose, 20 of g/L peptone, and appropriate concentration of ethanol and acetic acid.

### Acetic Acid Fermentation

For acetic acid fermentation, *A. pasteurianus* CGMCC 3089 was cultured in 500 ml Erlenmeyer flask with 100 ml of seed medium at 30°C and 180 r/min. When the optical density (OD) at 600 nm was approximately 1.2, the cells were transferred into a 5 L self-inspiriting fermenter (Nanjing Huike Bioengineering Equipment Corporation, Nanjing, China) containing 3.5 L of the fermentation medium. The start-up of acetic acid fermentation was performed under the following conditions: 30°C, aeration rate of 0.1 vvm (volume air per volume media per minute), and agitator speed of 2000 r/min with 7% (v/v) ethanol and 1% (w/v) initial acetic acid concentration. When the ethanol concentration fell to 0.5% a certain volume of broth was discharged, and the same volume of fresh medium was supplemented to start a semi-continuous fermentation. The DO electrode (Hamilton, Bonaduz, Switzerland) was calibrated to zero by using saturated solution of sodium sulphite, and the DO 100% was calibrated before inoculation under 30°C, 0.1 vvm and agitator speed of 2000 r/min.

The fermentation conditions of DO, initial acetic acid concentration and total concentration were optimized with semi-continuous fermentation technology. To study the effect of DO on the acetic acid and cell metabolism, the semi-continuous fermentations were performed at 10, 20 and 30% of the saturation DO, which present the low, middle, and high dissolved oxygen content according to the previous report (Romero et al., 1994). In the semi-continuous fermentation, the DO was controlled by adjusting the agitator speed and aeration rate.

The initial acetic acid concentration was optimized under the 90 g/L of total concentration and 20% DO. Initial acetic acid concentrations of 35, 40, and 45 g/L were obtained by discharging the fermentation broth of 60, 55 and 50%, respectively, and then fresh medium (containing about 90 g/L of ethanol) was supplemented.

For total concentration optimization, semi-continuous fermentations were performed with total concentration of 9, 10, and 11%, respectively, under 20% DO. For each batch of fermentation, appropriate fermentation broth was discharged and high ethanol medium was added to obtain the total concentration of 9% (4% acetic acid+5% ethanol), 10% (4% acetic acid +6% ethanol), and 11% (4% acetic acid +7% ethanol).

In this research, all experiments were repeated three times.

### Transcriptome Data Analysis and Metabolic Network Construction

For transcriptome analysis, 50 ml of broth was collected at OD_600_ = 1.2 from the start-up of acetic acid fermentation. Total RNA was isolated by using the RNA Plus Kit (Takara Biotechnology, Dalian, China) following the manufacturer’s procedure. To remove residual DNA, total RNA was treated with DNase I for 30 min at 37°C. RNA samples were reverse-transcribed with Revert Aid™ First Strand cDNA Synthesis Kit (Takara Biotechnology, Dalian, China) in accordance with the manufacturer’s instructions. Transcriptome sequencing was performed by Novogene Co., Ltd. (Tianjin). Quality control of the raw data generated by sequencing was carried out with FastQC (http://www.bioinformatics.babraham.ac.uk/projects/fastqc/). The transcriptome sequencing data has been uploaded to NCBI Short Read Archive and the accession number is SAMN17805140.

Gene expression level was calculated by fragment per kilo bases per million reads (FPKM) using RSEM software (v1.2.6) with 0.1 as the rounding threshold for gene expression. The gi numbers of the selected genes were imported into the Kyoto Encyclopedia of Genes and Genomes (KEGG) database (http://www.genome.jp/kegg/) for biological pathway analysis, with strain *A. pasteurianus* IFO3283-01 as reference (NC 013209.1). In particular, an energy metabolism network was constructed based on transcriptomic analysis, which mainly related to ethanol respiratory chain, glycolysis pathway, TCA cycle, amino acid metabolism, ATP production, and acetic acid tolerant proteins.

### Determination of Representative Genes Transcription in Fermentation

Several genes were selected to analyze the effect of fermentation conditions on the energy metabolism of *A. pasteurianus* by using the method of RT-PCR. Software of Primer 5 was used for primers design, which are listed in Table 1. Those are as following: *gnd* (encoding 6-phosphogluconate dehydrogenase, 6-PGD), *pyk* (encoding pyruvate kinase, PYK), and *ppdK* (encoding pyruvate phosphate dikinase, PPDK) involving in glucose metabolism; *cs* (encoding citrate synthase, CS), *mqo* (encoding malate: quinone oxidoreductase, MQO), *maeB* (encoding malic enzyme, ME), and *aarC* (encoding acetyl-CoA hydrolase, ACH) for the TCA cycle; *adh* (encoding alcohol dehydrogenase, ADH), and *cyto* (encoding cytochrome oxidase, CYTO) for the ethanol respiratory chain; *dnaK* (encoding molecular chaperone DnaK) relating to acid tolerance; and *atpB* (encoding ATP synthase *ß* subunit) for ATP generation.

**TABLE 1:** Primers used for RT-PCR.

Primers	Sequences (5′-3′)	Corresponding genes
16S-F	CCC​TTA​TGT​CCT​GGG​CTA​CA	16S rRNA
16S-R	CTC​ACC​GGC​TTA​AGG​TCA​AAC	
gnd-F	TGA​CCC​CAT​TCT​GAC​CTC​TCT	*gnd* (6-phosphogluconate dehydrogenase)
gnd-R	ACA​TCA​CGT​CAA​AGC​CTT​CC	
pky-F	GGC​ACA​CCT​ATT​GGC​ATT​CTG	*pyk* (pyruvate kinase)
pky-R	GCC​CTA​CTT​TTT​CCA​CCA​CAA​C	
ppdK-F	CTG​GAA​GAA​GTT​GCC​AAA​GC	*ppdK* (pyruvate phosphate dikinase)
ppdK-R	GTG​CCA​ATT​AGC​GGA​ATC​ATG	
cs-F	TTT​CAC​GTT​TGA​CCC​AGG​TT	*cs* (citrate synthase)
cs-R	GCA​GCA​GCG​TAT​GGT​TTG​TAA​G	
aarC-F	GCC​CGT​TTG​AAA​ATC​TGG​TAG	*aarC* (acetyl-CoA hydrolase)
aarC-R	GAC​TGT​TGC​TGA​CAT​CCT​GCT​G	
mqo-F	CGC​GTG​CAG​ATT​ATC​AAG​AA	*mqo* (malate: quinone oxidoreductase)
mqo-R	CTG​GCC​GTA​AGA​TGG​GAT​AA	
maeB-F	AGT​CTT​CCC​GCA​AAG​CTG​TA	*mae* (malic enzyme)
maeB-R	ACC​AGC​ATA​TCG​GCA​GTA​CCA	
atpB-F	AAA​CAC​TTG​GCC​GTA​TCC​TG	*atpB* (ATP synthase)
atpB-R	AAC​GGT​TTT​GCC​TAC​ACC​TG	
adh-F	CCA​AAA​CGC​ACC​TGG​TCT​AT	*adh* (alcohol dehydrogenase)
adh-R	TCT​TCC​AGA​CCG​TTT​CCA​TC	
dnaK-F	CCG​TTC​TGA​AGG​GTG​ATG​TTA	*dnaK* (molecular chaperone DnaK)
dnaK-R	TCG​AAG​TTA​CCC​AGC​AGC​TT	
Cyto-F	GTG​GCT​GCT​GGT​TGC​TGA​AGT​TGG​T	*cyto* (cytochrome oxidase)
Cyto-R	CGG​CAG​GTT​ACG​GTC​ACG​GAT​GGA​A	

Samples were collected at OD_600_ = 1.2 of each batch of fermentation. The total RNA was isolated using RNA Plus Kit as mentioned above. The quantity analysis of RNA was performed with ABI Step one system (Applied Biosystems, United States). The results were expressed by using 2^−△△Ct^ with the 16S rRNA as the internal standard gene. For each gene, the relative transcription of start-up fermentation was defined as the expression level of 1.0, and the result was expressed as the fold increase in mRNA compared with the control sample.

### Analytical Methods

The cell growth was monitored based on the OD_600_ value by a spectrophotometer (UVmini-1240, Shimadzu, Kyoto, Japan) at 610 nm. The biomass was measured according to the standard curves of dry cell weight (DCW) and OD value. The acetic acid content of the broth was determined by titration with 0.1 mol/L NaOH solution using phenolphthalein as an indicator. Glucose and ethanol contents were quantitated by suing a biosensor (Shan Dong academy of sciences, Jinan, China).

The experimental data for the specific rate (SR) of acetic acid production, ethanol consumption, or glucose consumption could be calculated with the following formula:

SR=dc/dt/Xt

where, SR is the specific rate of acetic acid production (g_acetic acid_/g _DCW_/h), ethanol consumption (g_ethanol_/g _DCW_/h), or glucose consumption (g_glucose_/g _DCW_/h). *d*_c_ */d*_*t*_ presents the rate of acetic acid production, ethanol consumption, or glucose consumption. *X*_t_ is the biomass at time t.

Intracellular ATP, ADP, and AMP were determined by kits (Ruishuo Biotechnology, Shanghai, China). EC was expressed by the formula EC = ATP+0.5ADP/([ATP] + [ADP] + [AMP]) (Chapman et al., 1971).

### Statistical Analysis

All experiments were repeated thrice. The results were expressed as the mean with standard error. The analysis of the differences between the categories was calculated with a confidence interval of 95% with SPSS (Least Significant Differences) analysis.

## 3 Results

### Correlation Between Acetic Acid Fermentation and Energy Charge

**FIGURE 1:**
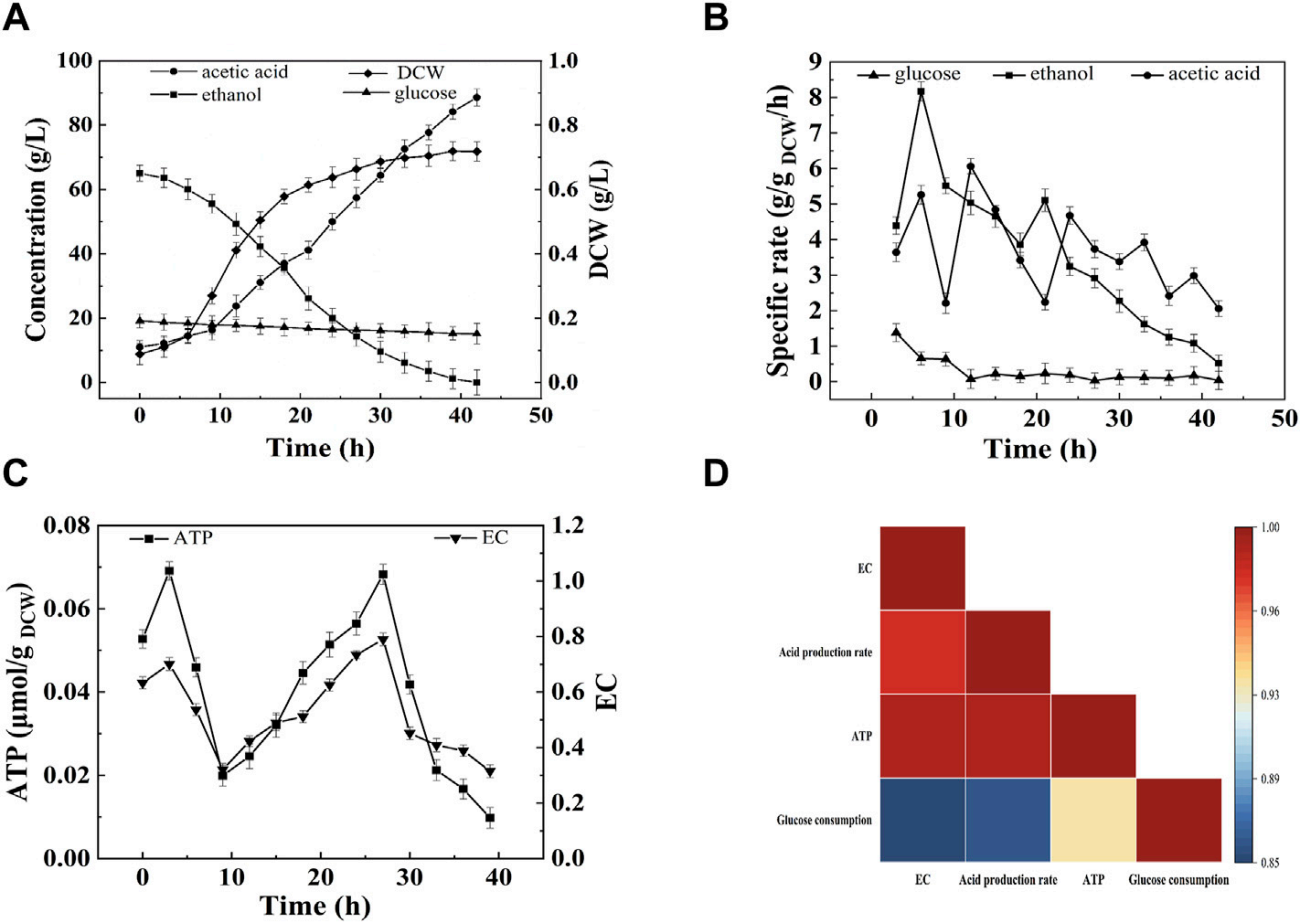
Time curves of start-up fermentation. **(A)** Acetic acid, ethanol, glucose concentration and cell dry weight; **(B)** Specific consumption rate of ethanol and glucose, and specific production rate of acetic a **(C)** EC and ATP; **(D)** Correlation analysis. The start-up of acetic acid fermentation was performed under 30°C, 0.1 vvm with 7% (v/v) ethanol and 1% (w/v) initial acetic acid.

As described in Figure 1A, the acetic acid production and cell growth corresponded with the consumption of ethanol and glucose. At the end of fermentation, the terminal acid concentration and the average acid production rate were 88.60 g/L and 2.06 g/L/h, respectively. However, the SR of acetic acid production and ethanol, and glucose consumption decreased with the increase of acetic acid (Figure 1B). Interestingly, there were two peaks for the intracellular ATP content and EC (Figure 1C), indicating the change of energy metabolism due to the change of fermentation conditions (Zheng et al., 2017). The high ATP content and EC were consisted with the high SR of acid production and ethanol consumption. In the initial stage of fermentation (0–9 h) ATP content and EC increased and then decreased. During this period, both of ethanol and glucose were used as the energy source. The average energy production rate from ethanol and glucose were 11.24 and 3.68 kJ/L/h, respectively. After 9 h of fermentation, ethanol oxidation became the main source for energy production (Figure 1B). The intracellular ATP content and EC increased, and acetic acid was produced rapidly (Figure 1A). From 9 to 27 h, the average ethanol oxidation rate was 2.293 g/L/h, and the average energy production rate from ethanol and glucose were 24.54 and 1.36 kJ/L/h, respectively. After 27 h, the SR of ethanol consumption decreased, and the ATP content and EC decreased (Figure 1C).

Correlation analysis was conducted to reveal the relationship between EC and acetic acid fermentation. As shown in Figure 1D, the acetic acid production rate was highly correlated with intracellular EC (*p* < 0.01) and ATP (*p* < 0.01), but lowly correlated with glucose consumption (*p* > 0.05). The correlation between glucose consumption and EC or ATP was also low. These results suggested that intracellular EC and ATP content might be important indexes for acetic acid fermentation.

### Transcriptome Analysis and Construction of Energy Metabolic Pathway

Transcriptome analysis was performed to elucidate the energy metabolism of *A. pasteurianus* in acetic acid fermentation. RNA sequencing yielded more than 2 G of data with a high degree of matching and an error rate of less than 1% (as listed in Supplementary Table S1).

**FIGURE 2:**
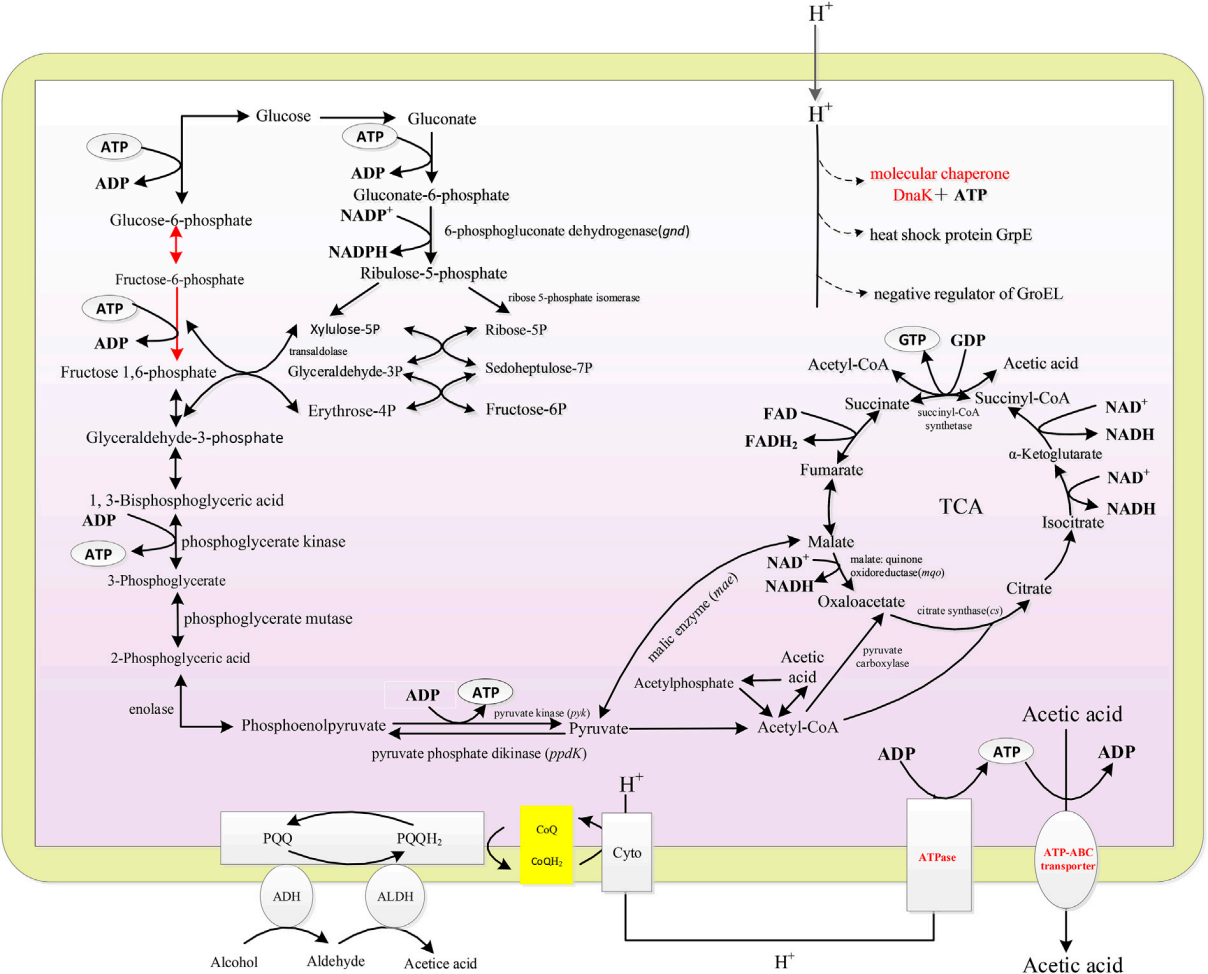
The scheme of energy metabolic and acetic acid tolerance related pathways of *A. pasteurianus*.

Gene Ontology enrichment was analyzed using GOseq. The enriched genes were mainly categorized into four groups, namely, ribosome metabolism, glyoxylate and dicarboxylate metabolism, microbial metabolism in diverse environments, and carbon metabolism. The main energy metabolism pathway of *A. pasteurianus* in acetic acid fermentation was constructed according to KEGG analysis, which are all related to the acetic acid fermentation or acetic acid tolerance of AAB. As described in Figure 2, *A. pasteurianus* produces energy mainly through the ethanol respiratory chain, TCA cycle, and PPP pathway. Through the ethanol respiratory chain, ethanol is oxidized to acetic acid by membrane bound ADH and ALDH. The electrons lost in the process of ethanol oxidation can be absorbed by the free shuttle coenzyme Q on the membrane, and the reduced coenzyme Q is oxidized by terminal oxidase (cytochrome oxidase, CYTO). Four H^+^ combine with one molecule of oxygen, and two molecules of water are generated to complete the whole ethanol respiration. Meanwhile, ATP is produced by ATPase (González et al., 2006; Trcek et al., 2015). Through the glycolysis pathway, one molecule of glucose produces two molecules of pyruvate and two ATP under the action of 3-phosphoglyceraldehyde dehydrogenase, phosphoglycerate kinase, phosphoglycerate mutase, and phosphopyruvate hydratase. Owing to the lack of phosphate fructose kinase, PPP pathway is the main glucose consumption pathway of *A. pasteurianus* (Azuma et al., 2009; Sakurai et al., 2011; Illeghems et al., 2013). In the PPP pathway, glucose is converted into ribulose consequently by 6-PGD, transaldolase, and 5-phosphate ribose isomerase. PYK uses phosphoenolpyruvate to produce pyruvate and ATP. PPDK can convert pyruvate to phosphoenolpyruvate, which involved in gluconeogenesis. TCA cycle is important for the energy production and acetic acid tolerance of *A. pasteurianus* (Zheng et al., 2018b). CS, MQO, ME, and ACH are all related to the acetic acid tolerance of AAB (Fukaya et al., 1990; Fukaya et al., 1993; Mullins et al., 2008). In addition, some stress response proteins, including ATP-binding transporter, molecular chaperone DnaK and GroEL, are related to the acetic acid tolerance of *A. pasteurianus* (Susin et al., 2006; Andrés-Barrao et al., 2016).

### Effects of Fermentation Conditions on the Energy Metabolism

Semi-continuous acetic acid fermentation was performed after the start-up of fermentation. The effects of fermentation conditions, including DO, initial acetic acid concentration and total concentration, on the cell energy metabolism and acetic acid fermentation were analyzed.

#### Effect of DO on the Energy Metabolism and Acetic Acid Fermentation

**FIGURE 3:**
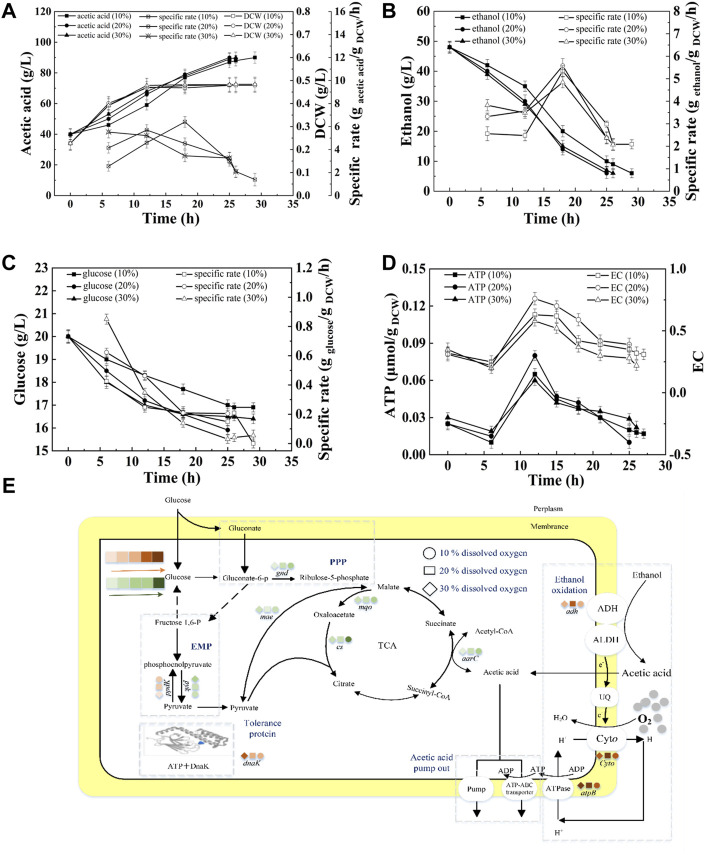
Effect of DO on the acetic acid fermentation. **(A)** Concentration and specific production rate of acetic acid, and DCW; **(B)** Concentration and specific consumption rate of ethanol; **(C)** Concentration and specific consumption rate of glucose; **(D)** EC and ATP; **(E)** The scheme of energy metabolic pathway. Orange represents up-regulation of gene transcription, and green represents down-regulation.The effect of DO on the fermentation was compared with the 90 g/L of total concentration and 40 g/L of initial acetic acid, under 10, 20, and 30% DO, respectively.

As described in Figure 3A, under 10, 20, and 30% DO, the fermentation periods were 29, 25, and 26 h, respectively. The average acid production rate under 10% DO was the lowest (1.72 g/L/h). No significant difference in the average acid production rates was found between 20 and 30% DO (*p* < 0.5), which were 2.00 g/L/h and 1.92 g/L/h, respectively (Supplementary Table S2). The highest SR of ethanol consumption was obtained at 18 h, when the cell was at the late-logarithmic growth phase (Figure 3B). The SR of glucose consumption decreased (Figure 3C). Those results were agreed with the start-up fermentation. The highest SR of ethanol consumption, intracellular EC and ATP and the shortest fermentation period were all obtained at 20% DO, indicating 20% DO was sufficient for ethanol oxidation and energy generation. This result was agreed with the high correlation of acetic acid production and EC or ATP (Figure 1D). Although acetic acid fermentation is an aerobic process, more than about 30% of saturation DO might has an inhibiting effect on cell growth (Romero et al., 1994).

The transcription of genes related to the energy metabolism and acetic acid tolerance was analyzed with the method of RT-PCR. ADH and CYTO are important for ethanol oxidation. As described in Figure 3E, the transcription levels of *adh* and *ctyo* increased with increasing DO from 10 to 20%. However, they were no significant different at 20 and 30% DO. The enhanced expression of *adh* promoted the acetic acid production, and improved the electron transfer and energy generation. Protein AtpB is related to ATP production. The highest transcription of AtpB corresponded to the highest intracellular ATP concentration as shown in Figure 3D. Molecular chaperone DnaK was up-regulated with increasing DO (as described in Supplementary Figure S1). 6-PGD encoded by gene *gnd* is the rate-limiting enzyme of the pentose phosphate pathway (Tian et al., 1998). PYK utilizes phosphoenolpyruvate to generate pyruvate and ATP. PPDK is involved in gluconeogenesis, which can convert pyruvate into phosphoenolpyruvate. The expression of *gnd* and *pyk* were down-regulated during semi-continuous fermentation when compared with that of start-up fermentation, while *ppdK* increased (Supplementary Figure S1), indicating the inhibited glucose metabolism. TCA cycle is negatively correlated with acetic acid fermentation (Vuong et al., 2005; Sakurai et al., 2012). The expressions of *cs* and *mqo* in semi-continuous fermentation were all down-regulated compared with those of start-up fermentation. Therefore, ethanol oxidation became the main source for energy generation in semi-continuous fermentation instead of TCA cycle.

#### Effect of Initial Acetic Acid Concentration on the Energy Metabolism and Acetic Acid Fermentation

**FIGURE 4:**
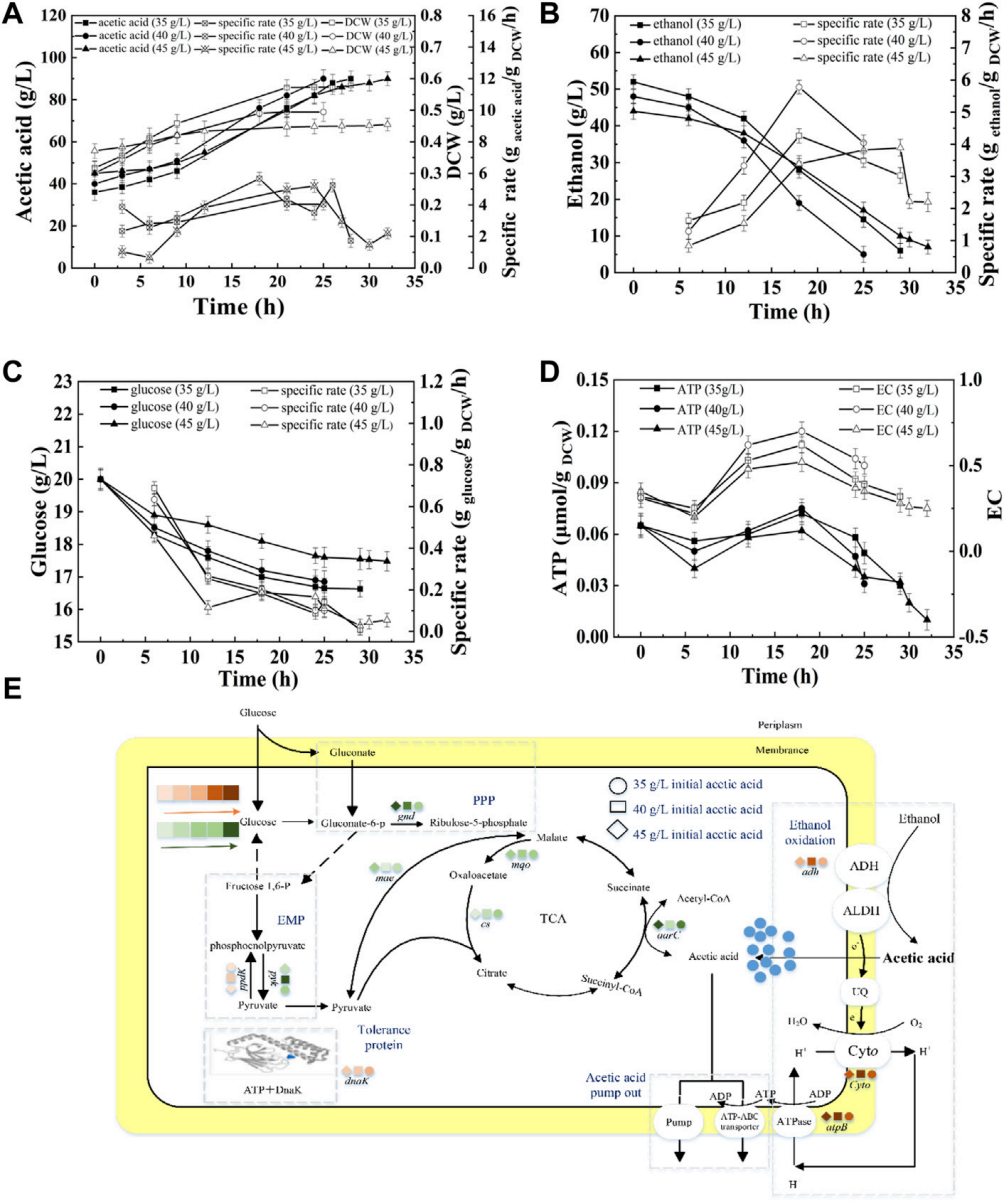
Effect of initial acetic acid on the acetic acid fermentation. **(A)** Concentration and specific production rate of acetic acid, and DCW; **(B)** Concentration and specific consumption rate of ethanol; **(C)** Concentration and specific consumption rate of glucose; **(D)** EC and ATP; **(E)** The scheme of energy metabolic pathway. Orange represents up-regulation of gene transcription, and green represents down-regulation.The effect of initial acetic acid concentration on the fermentation was compared with 20% DO, 90 g/L of total concentration, under 35, 40, and 45 g/L of initial acetic acid concentration, respectively.

Figure 4A shows that when the concentration of initial acetic acid was 35 g/L, 40 g/L, or 45 g/L, the average acetic acid production rate was 1.90 g/L/h, 2.00 g/L/h, or 1.41 g/L/h, respectively (Supplementary Table S3). The highest SR of ethanol consumption was obtained at 40 g/L initial acetic acid (Figure 4B). The high SRs of ethanol consumption were at 17–24 h, accompanying with the high intracellular EC and ATP (Figure 4D). However, at 45 g/L of initial acetic acid, the cell growth and ethanol oxidation were inhibited. The terminal DCW were 0.579, 0.495, and 0.455 g _DCW_/L, at 35, 40, and 45 g/L initial acetic acid, respectively, indicating the increase of inhibition with increasing acetic acid.

As described in Figure 4E, the highest transcription levels of *adh* and *ctyo* were obtained under 40 g/L of initial acetic acid. The expression of *gnd* was down-regulated with increasing initial acetic acid concentration, indicating that glucose metabolism was inhibited. Moreover, the lowest expression of *pyk* and highest expression of *ppdK* suggested that the lowest energy produced by glucose metabolism at 40 g/L of initial acetic acid. ME can catalyze pyruvate to supplement the deficiency of the TCA cycle. The highest transcription of gene *mae* was achieved at 40 g/L of initial acetic acid. The up-regulated expression of ME would promote the flow of pyruvate into the TCA cycle and the glycolysis pathway. The *aarC* gene had the highest transcription level under 40 g/L of initial acetic acid, indicating that additional acetyl-CoA was produced and flowed into the TCA cycle. In addition, the transcription of *atpB* under 40 g/L of initial acetic acid was the highest when compared the others. Therefore, the highest intracellular EC and ATP content were obtained under 40 g/L of initial acetic acid (Figure 4D). The expression of molecular chaperone DnaK was up-regulated with increasing in initial acetic acid concentration (Supplementary Figure S2).

#### Effect of Total Concentration on the Energy Metabolism and Acetic Acid Fermentation

**FIGURE 5:**
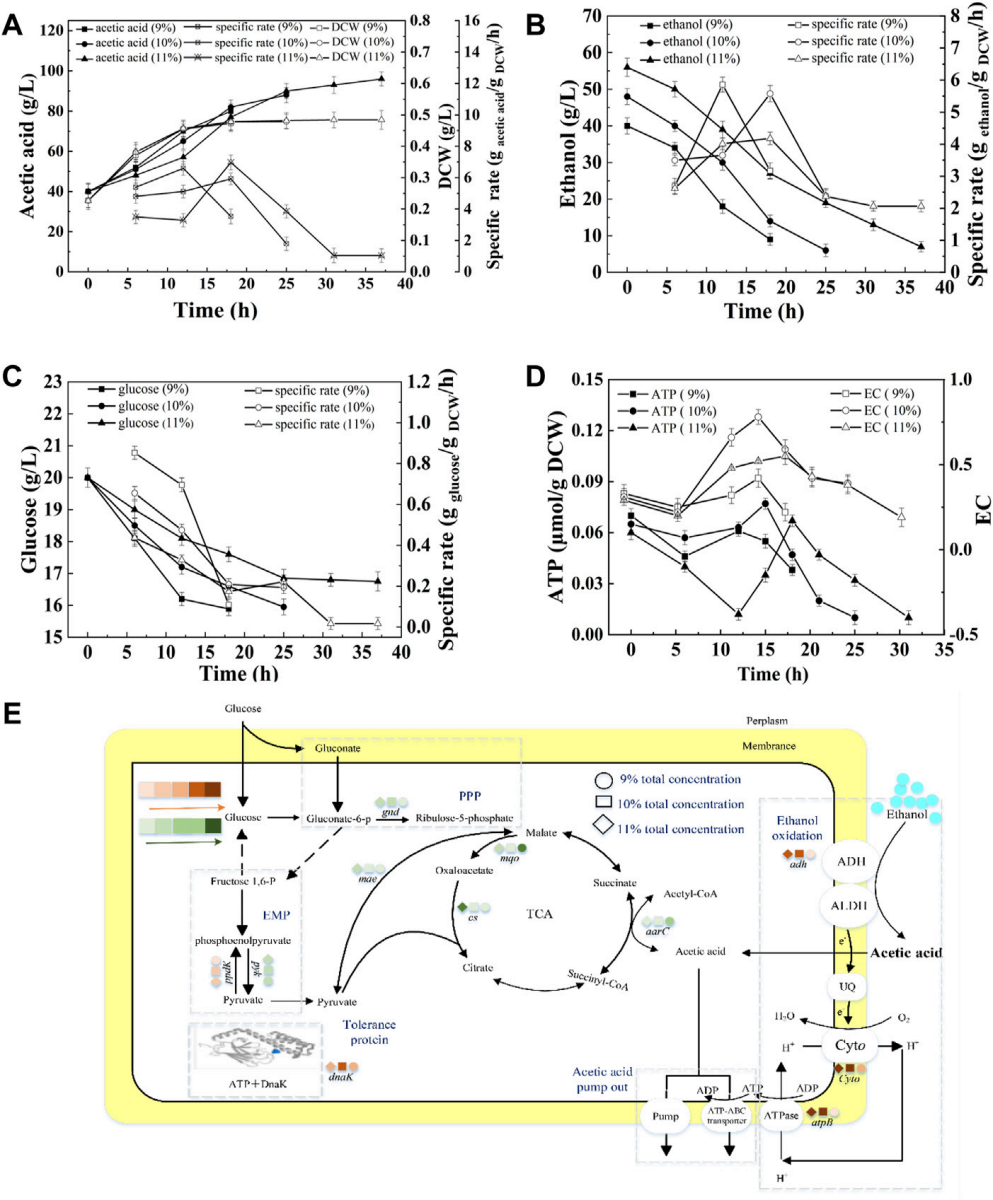
Effect of total concentration on the acetic acid fermentation. **(A)** Concentration and specific production rate of acetic acid, and DCW; **(B)** Concentration and specific consumption rate of ethanol; **(C)** Concentration and specific consumption rate of glucose; **(D)** EC and ATP; **(E)** The scheme of energy metabolic pathway. Orange represents up-regulation of gene transcription, and green represents down-regulation.The effect of total concentration on the fermentation was compared with 20% DO, 40 g/L of initial acetic acid, under 90, 100, and 110 g/L of total concentration, respectively.

Ethanol and acetic acid are the substrate and product of acetic acid fermentation, respectively. AAB convert ethanol into acetic acid, and the total concentration in the fermentation broth remains almost unchanged (except for the amount lost due to volatilization). Therefore, the total concentration has an important effect on acetic acid fermentation. Figure 5A shows that at total concentrations of 9, 10, and 11%, the fermentation periods were 18, 25, and 37 h, respectively, and the average acetic acid production rates were 2.22, 1.92, and 1.53 g/L/h (Supplementary Table S4). In the initial stage of fermentation (0–12 h), the SRs of acetic acid production and glucose consumption decreased with increasing total concentration (Figures 5A,C). The terminal biomasses were similar, however, the SRs of acetic acid production and ethanol consumption at the terminal time decreased with increasing total concentration, indicating the inhibition effect of total concentration on acetic acid fermentation (Figures 5A,B). The concentrations of initial acetic acid in three fermentations were the same (40 g/L), thus the inhibition effect was mainly due to the high concentration of ethanol in the beginning of fermentation and the high acidity in the late stage of fermentation. As shown in Figure 5E, the highest expression of *adh*, *cyto*, *atpB*, and *dnak* were achieved under10% total concentration, which produced the highest intracellular EC and ATP content (Figure 5D). The expression of *gnd* down-regulated and *ppdK* up-regulated with increasing total concentration, thereby resulting in the decreased RS of glucose consumption (Figure 5C).

## Discussion

Acetic acid was industrially fermented with AAB with ethanol as substrate. Ethanol is oxidized by membrane-bound ADH and ALDH, and the product acetic acid remains extracellular. Acetic acid is toxic to bacteria. The toxic effects of acetic acid on microorganisms include the reduction of intracellular pH and metabolic disorders caused by anion accumulation (Trcek et al., 2015; Zheng et al., 2017). When pH is lower than its p*Ka* value (4.75) most acetic acid molecule is protonated, and protonated acetic acid can permeate into microbial cells by passive diffusion and dissociating directly into microbial cytoplasm, thereby decreasing intracellular pH and anions, destroying the transmembrane proton gradient, and inducing metabolic uncoupling (Konings et al., 1989; Andrés-Barrao et al., 2012; Trcek et al., 2015; Li et al., 2019). Intracellular acidification inhibits the activity of glycolytic enzymes, leading to cellular energy deficiency (Pampulha and Loureiro-Dias, 1990). To resist acetic acid stress AAB up-regulate the defense mechanism which consumes energy (Mira et al., 2010). Ethanol respiratory chain and glucose oxidation are two main energy generation pathways in *A. pasteurianus*. AAB can maintain reasonable energy metabolism for cell growth by improving ethanol oxidation instead of glucose metabolism, therefore accelerating the acetic acid production (Zheng et al., 2017). Ethanol oxidation were highly correlated with EC (as described in Figure 1D). EC can reflect the energy status of bacteria and regulate cell metabolism. In beer fermentation EC is related with the ethanol tolerance of yeast, and there is high correlation between maintenance of the EC and active transport of *a*-glucosides (Guimaraes and Londesborough 2008). In prokaryote *Escherichia coli*, EC regulates signal transduction proteins, metabolic enzymes, and permeases involved in assimilation, therefore regulating the metabolic rates (Andersen 1977; Jiang and Ninfa 2009). In this work, the effects of fermentation conditions, including DO, initial acetic acid concentration, and total concentration, on the acetic acid fermentation and the energy metabolism of *A. pasteurianus* were analyzed. The results showed that a virtuous circle of increased ethanol oxidation, increased energy generation, and acetic acid tolerance was important for improving acetic acid fermentation.

As described in Figure 2, energy metabolism is important for cell growth and metabolism and is positively related to acetic acid fermentation. Qi et al. (2014b) increased the energy metabolism of *A. pasteurianus* by adding some precursors of ethanol respiratory chain related factors (ferrous ions andβ-hydroxybenzoic acid) to improve the acetic acidization. However, the regulation of energy metabolism in acetic acid fermentation is unclear. In acetic acid fermentation, the EC of the cells dynamically changed due to the production and consumption of ATP (Figure 1C). With the ethanol oxidization, the H^+^ in the oxidized ubiquinone (UQ) is released into the periplasm thereby producing the proton motive force required to generate energy (Wang Z. et al., 2015). Meanwhile, the proton motive force is used to pump acetic acid out of the cell to achieve the acetic acid tolerance (Matsushita et al., 2005). The up-regulated expression of *adh* and *cyto* enhanced ethanol respiratory chain, which in turn improved the energy metabolism. High EC promotes anabolism and inhibits catabolism (Adler et al., 2014). The enzymes involved in the TCA cycle were down-regulated probably due to the high EC level produced from ethanol oxidation. The decreased TCA cycle and the improved ethanol respiratory chain made ethanol oxidation the main energy generation pathway in acetic acid fermentation. Therefore, the highest SR of ethanol oxidation was achieved when EC was the highest lever, which was agreed with the result (Figures 3B,D, 4B,D, 5B,D).

Although most of the ethanol is oxidized out of the cell, some can enter the cell and be oxidized into acetic acid by intracellular ADH (Trcek et al., 2006; Saichana et al., 2015; Wang et al., 2015b; Wang et al., 2015c). Acetic acid may enter the TCA cycle through ACH to strengthen the energy metabolism, thereby producing reducing power and ATP to meet the energy needs for cell growth and acetic acid tolerance (Lasko et al., 2000; Mullins et al., 2008). This phenomenon explains the high correlation between AarC expression levels and intracellular ATP and EC (as described in Figures 3D,E, 4D,E, 5D,E). In addition to enzymes from some metabolism pathways, the expression of AtpB was highly correlated with the highest EC and energy metabolism. The expression of molecular chaperone DnaK was related to the energy metabolism because ATP consumption is needed to ensure the correct folding of protein under acid pressure (Hartl and Hayer-Hartl 2002; Ishikawa et al., 2010).

## Data Availability

The original contributions presented in the study are publicly available. This data can be found here: https://www.ncbi.nlm.nih.gov/bioproject/, PRJNA699689.
