# Supplementary material for: Improving the Acetic Acid Fermentation of Acetobacter pasteurianus by Enhancing the Energy Metabolism
Source: Front Bioeng Biotechnol. 2022 Mar 8;10:815614. doi: 10.3389/fbioe.2022.815614 (PMC8957916; doi:10.3389/fbioe.2022.815614)

Supplementary Material

# Supplementary Tables and Figures

**Table S1** **Quality of data output**

| Raw reads | Clean reads | Clean bases | Error rates (%) | Q20  (%) | Q30  (%) | GC content (%) |
| --- | --- | --- | --- | --- | --- | --- |
| 14808050 | 14552396 | 2.1 G | 0.03 | 98.63 | 94.27 | 53.61 |

**Raw reads:** Count the raw sequence data, and count the number of sequencing sequences in each file in four lines. **Clean reads:** The calculation method is the same as Raw Reads, except that the statistical files are filtered sequencing data. The subsequent analysis of biological information is based on Clean reads. **Clean bases:** The number of Clean reads is multiplied by the length, and converted into units of G. **Error rate:** calculated by Equation, Qphred = -10log10(e), the phred value is calculated by a probability model during the base calling process, which can accurately predict the error rate of base discrimination. **Q20, Q30:** Calculate the percentage of bases with Phred value greater than 20 and 30 respectively to the total bases. **GC content:** Calculate the percentage of the total number of bases G and C as a percentage of the total number of bases.

**Table S2 Effect of DO on acetic acid fermentation**

| DO (%) | Fermentation period (h) | Final acidity (g/L) | *R_acid_*  (g/L/h) | Final biomass  (g/L) | *R_biomass_*  (g/L/h) |
| --- | --- | --- | --- | --- | --- |
| 10 | 29 | 90 | 1.72±0.02 | 0.476±0.01 | 0.0164±0.009 |
| 20 | 25 | 90 | 2.00±0.01 | 0.481±0.013 | 0.0192±0.011 |
| 30 | 26 | 90 | 1.92±0.07 | 0.484±0.02 | 0.0186±0.019 |

*R_acid_* represents the average acetification rate. *R_biomass_* represents the average cell growth rate.

**Table S3 Effect of initial acetic acid on acetic acid fermentation**

| Initial acetic  acid (g/L) | Fermentation period (h) | Final acidity  (g/L) | *R_acid_*  (g/L/h) | Final biomass  (g/L) | *R_biomass_*  (g/L/h) |
| --- | --- | --- | --- | --- | --- |
| 35 g/L | 29 | 90 | 1.90±0.01 | 0.579±0.013 | 0.0199 ± 0.002 |
| 40 g/L | 25 | 90 | 2.00±0.09 | 0.495±0.024 | 0.0198±0.003 |
| 45 g/L | 32 | 90 | 1.41±004 | 0.455±0.038 | 0.0142±0.004 |

*R_acid_* represents the average acetification rate. *R_biomass_* represents the average cell growth rate.

**Table S4 Effect of total concentration on acetic acid fermentation**

| Fermentation period (h) | Total concentration* (v/v) | *R_acid_* (g/L/h) | Final biomass (g/L) | *R_biomass_*  (g/L/h) |
| --- | --- | --- | --- | --- |
| 18 | 9 (4%A＋5%E) | 2.22±0.02 | 0.473±0.011 | 0.0263±0.0001 |
| 25 | 10 (4%A＋6%E) | 1.92±0.04 | 0.478±0.012 | 0.0191±0.0012 |
| 37 | 11 (4%A＋7%E) | 1.53±0.01 | 0.484±0.011 | 0.0131±0.0015 |

*A presents acetic acid, and E presents ethanol. *R_acid_* represents the average acetification rate. *R_biomass_* represents the average cell growth rate.

**Figure S1 The changes of gene transcription in *A. pasteurianus*** **under different DO**

**（A）Glucose metabolism （B）TCA cycle**

**（C）Ethanol oxidation （D）ATP production and anti-stress protein**

**
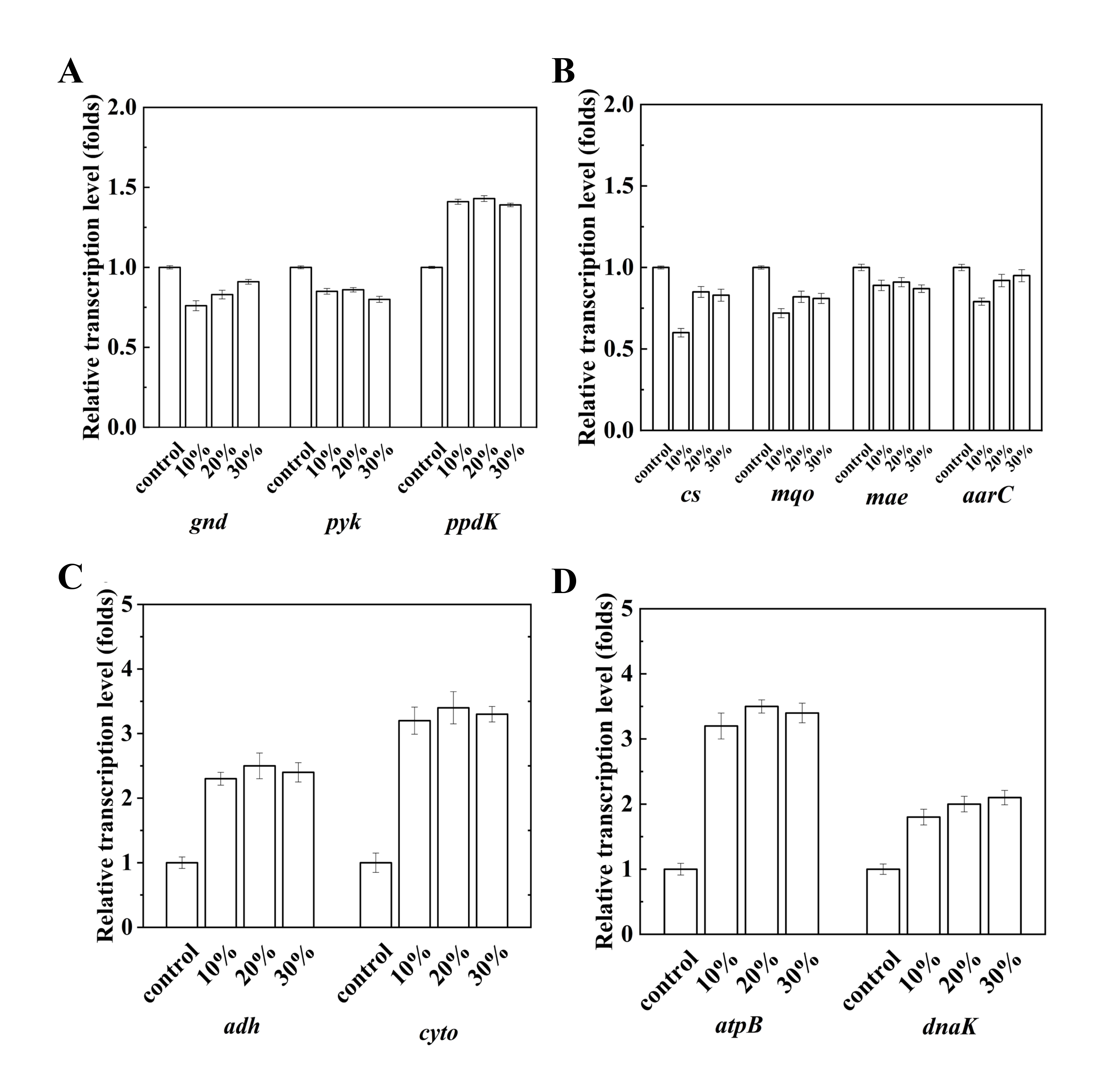
**

**Figure S2 The change of genes transcription in *A. pasteurianus* under different initial acetic acid concentration**

**（A）Glucose metabolism（B）TCA cycle**

**（C）Ethanol oxidation （D）ATP production an anti-stress protein**

**
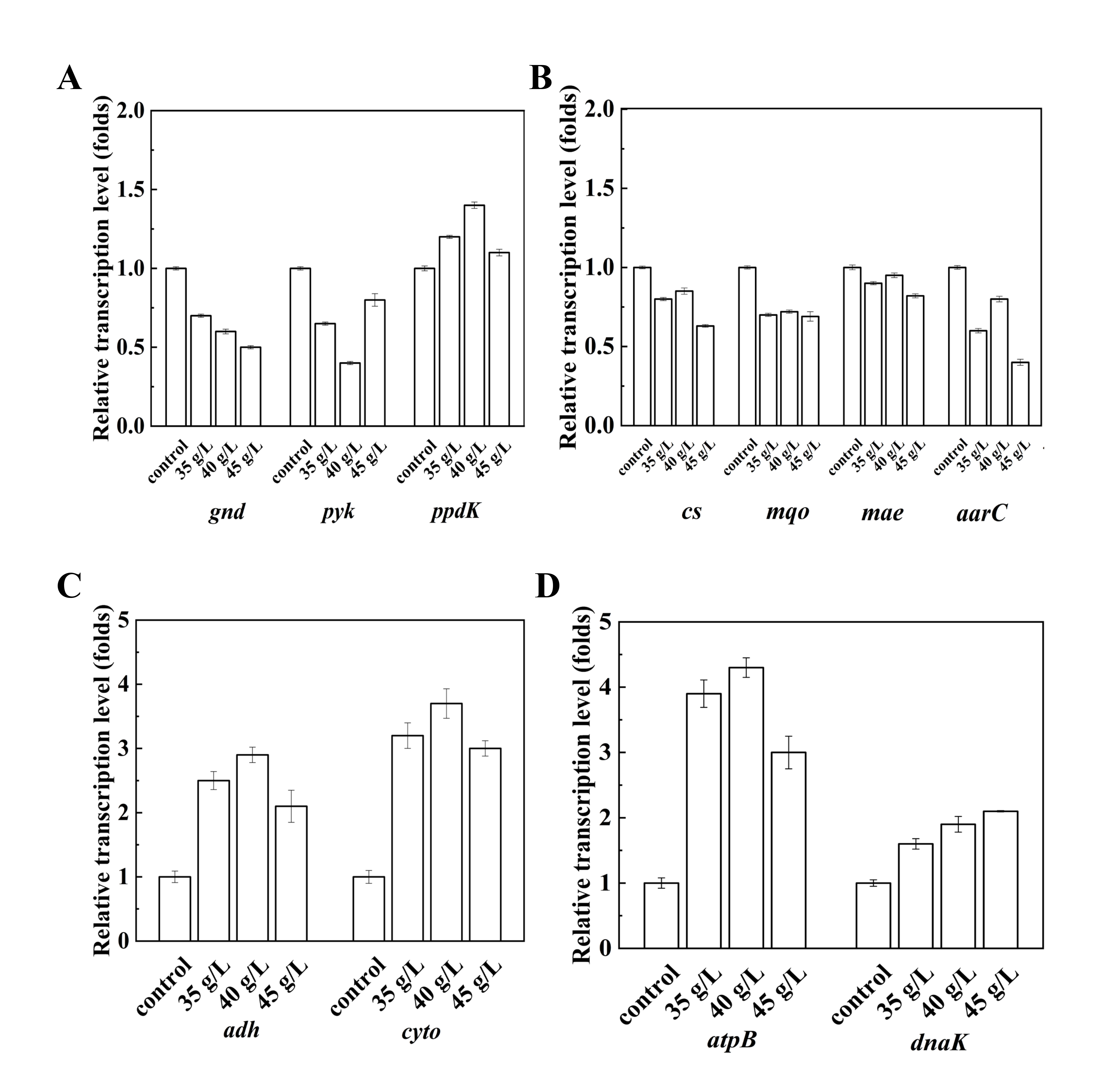
**

**Figure S3 The change of genes transcription in *A. pasteurianus* under different total concentration**

**（A）Glucose metabolism （B）TCA cycle**

**（C）Ethanol oxidation （D）ATP production and anti-stress protein**


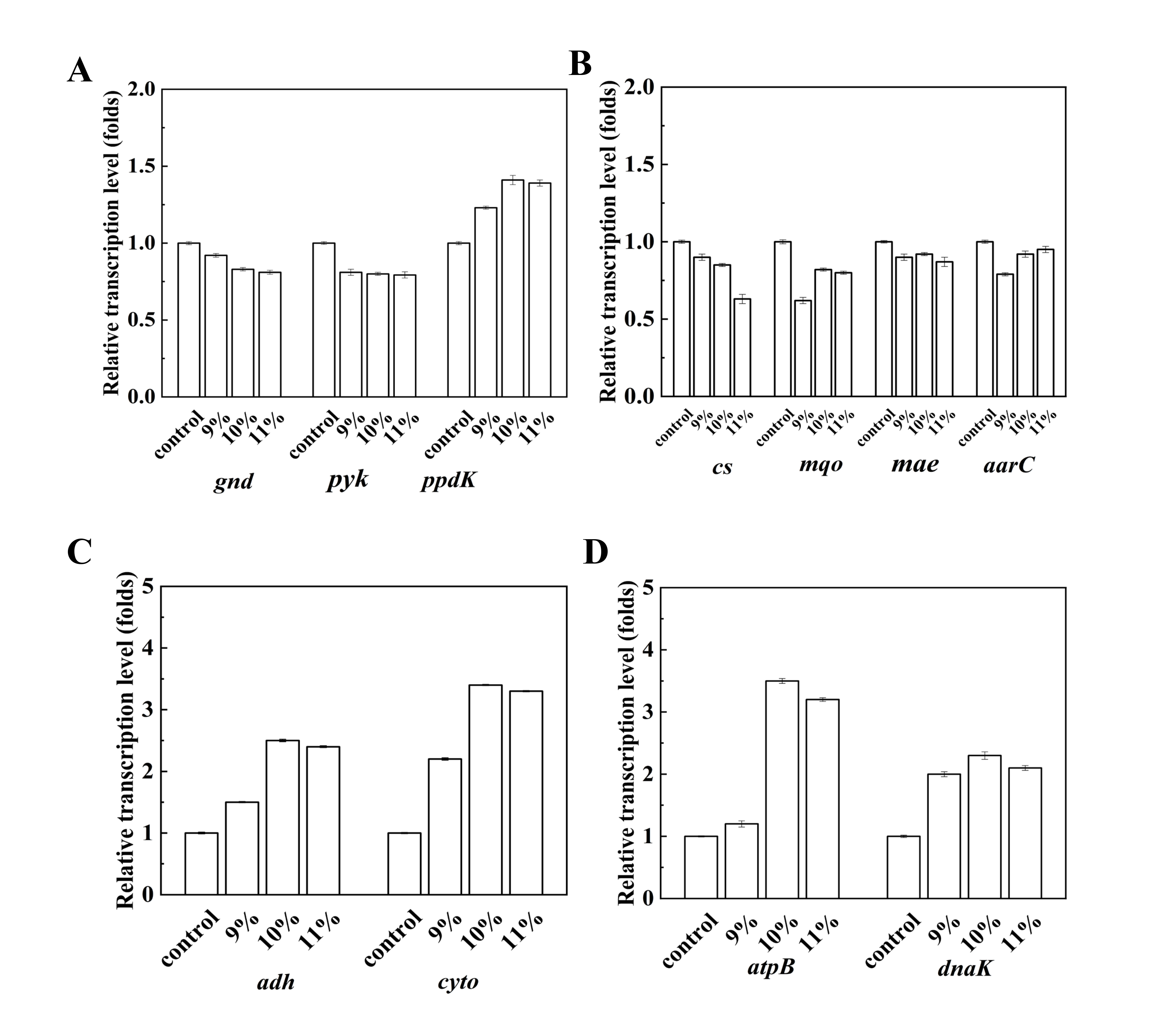

Supplement: Supplementary file 1 [file DataSheet1.doc]
